# Sequential MRI in pontine and extrapontine myelinolysis following rapid correction of hyponatremia

**DOI:** 10.1186/s13104-018-3816-5

**Published:** 2018-10-05

**Authors:** Robert Laureno, Guillaume Lamotte, Alexander S. Mark

**Affiliations:** 1 Department of Neurology, Medstar Washington Hospital Center, Washington, DC USA; 2 Department of Neurology, Medstar Georgetown University Hospital, 3800 Reservoir Road NW, Washington, DC 20007 USA; 3 Department of Radiology, Medstar Washington Hospital Center, Washington, DC USA; 4 Bethesda MRI & CT, Rockville, MD USA

**Keywords:** Myelinolysis, Hyponatremia, MRI, Osmotic demyelination

## Abstract

**Objective:**

This study describes the MRI changes associated with pontine and extrapontine myelinolysis secondary to rapid correction of hyponatremia in dogs. The authors discuss the relevance of the results for theories of pathogenesis and for diagnosis of patients.

**Results:**

MRI changes associated with pontine and extrapontine myelinolysis first occur on diffusion-weighted imaging. As a generalization, gadolinium enhancement, flair image change and T_2_ weighted image abnormality appear sequentially.

## Introduction

Pontine and extrapontine myelinolysis can occur after the rapid correction of hyponatremia. In this disease, myelin and oligodendrocytes are damaged, while the neurons and axons are relatively spared [1]. The lesions occur symmetrically in the thalamus, pons, striatum, cerebral cortex, cerebellum and other areas [2]. Common clinical manifestations of myelinolysis are paralysis, dysarthria and obtundation [3].

Demonstration of myelinolysis by MRI has been studied retrospectively [4]. In humans, prospective study of the disease by frequent, sequential scans is not practical. However, such repeated scanning can be done in animals. For MRI study we chose canine myelinolysis, the animal model which best resembles the human disease both clinically and pathologically [5]. In our initial study we have shown that experimental myelinolysis in dogs occurs after rapid correction of hyponatremia but not after uncorrected hyponatremia [5]. Adding to that prior clinicopathologic study we now report sequential MRI findings of the canine disease. There follows a discussion of the results and their relevance for both theories of pathogenesis and diagnosis of patients.

## Main text

### Methods

The method of inducing myelinolysis has been described elsewhere [5]. Hyponatremia was maintained for a minimum of 3 days. The day of infusion of 3% saline to correct hyponatremia is considered day 0. As a rule, sequential daily MRI brain scans were performed on day 1 until death or sacrifice of the animal [5]. Sacrifice was performed with intravenous pentobarbital [5]. Intramuscular injection of an acepromazine and ketamine mixture (1.5-5 cc) sedated the dogs for scanning. A 1.5 Tesla Philips Gyroscan ACS-NT was used. T_1_ weighted, T_2_ weighted, Flair and Diffusion weighted images were obtained as were T1 weighted images after intravenous gadolinium infusion. Clinical observations were made daily. Autopsy was performed on selected animals in the manner previously described [5]. The Institutional Review Board at Washington Hospital Center approved the study and the experiments were performed according to the hospital guidelines.

### Results

Induction of hyponatremia was attempted in 18 dogs. Only seven were included in the study. Eleven were excluded because of failure to achieve hyponatremia (4 dogs), death during induction of hyponatremia (5 dogs) or death during the first 24 h after the hypertonic saline infusion (2 dogs).

In the seven study dogs, the precorrection sodium levels ranged from 99 to 127 mmol/L (only one dog had a precorrection serum sodium above 118). At 24 h after hypertonic saline infusion, the rise in sodium ranged from 20 to 26 mmol/L.

The clinical deterioration observed after correction of hyponatremia ranged from altered alertness to stupor and from trouble walking to quadriparesis. One of the 7 dogs showed no clinical deterioration. On average, neurologic deterioration first became evident on day 3.6 (range day 2–6). In 4 of the 7 dogs, the MRI scans showed myelinolysis before or in the absence of neurologic change. On the other hand, 3 dogs showed neurologic worsening before the appearance or in the absence of imaging abnormalities.

**Fig. 1:**
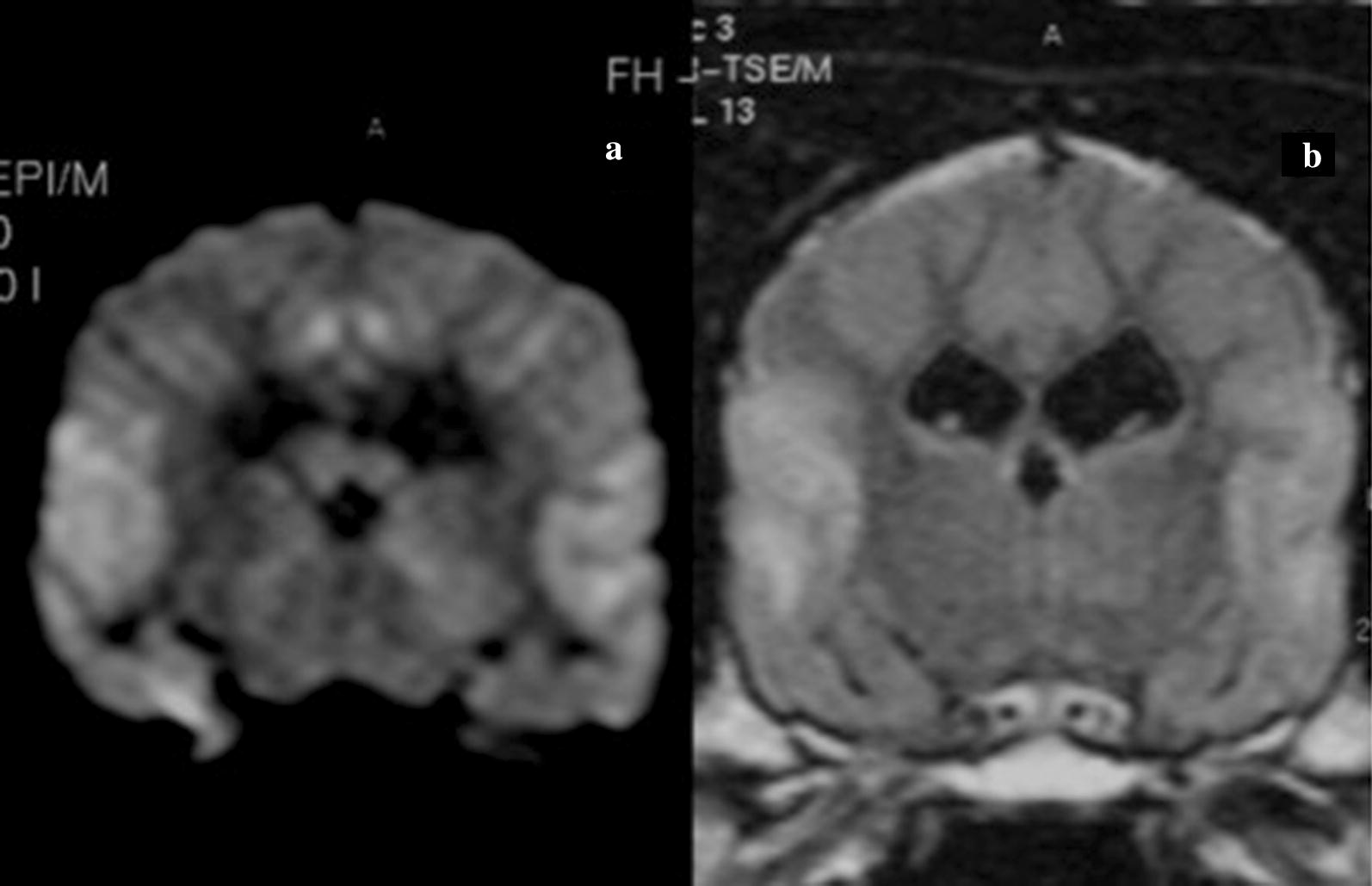
Diffusion weighted (**a**) and flair (**b**) images on the same day reveal hyperintensities of the opercular cortex bilaterally. Diffusion weighted images (**a**) reveal additional bilateral, parasagittal, cortical areas of hyperintensity which are not seen on flair images

**Fig. 2:**
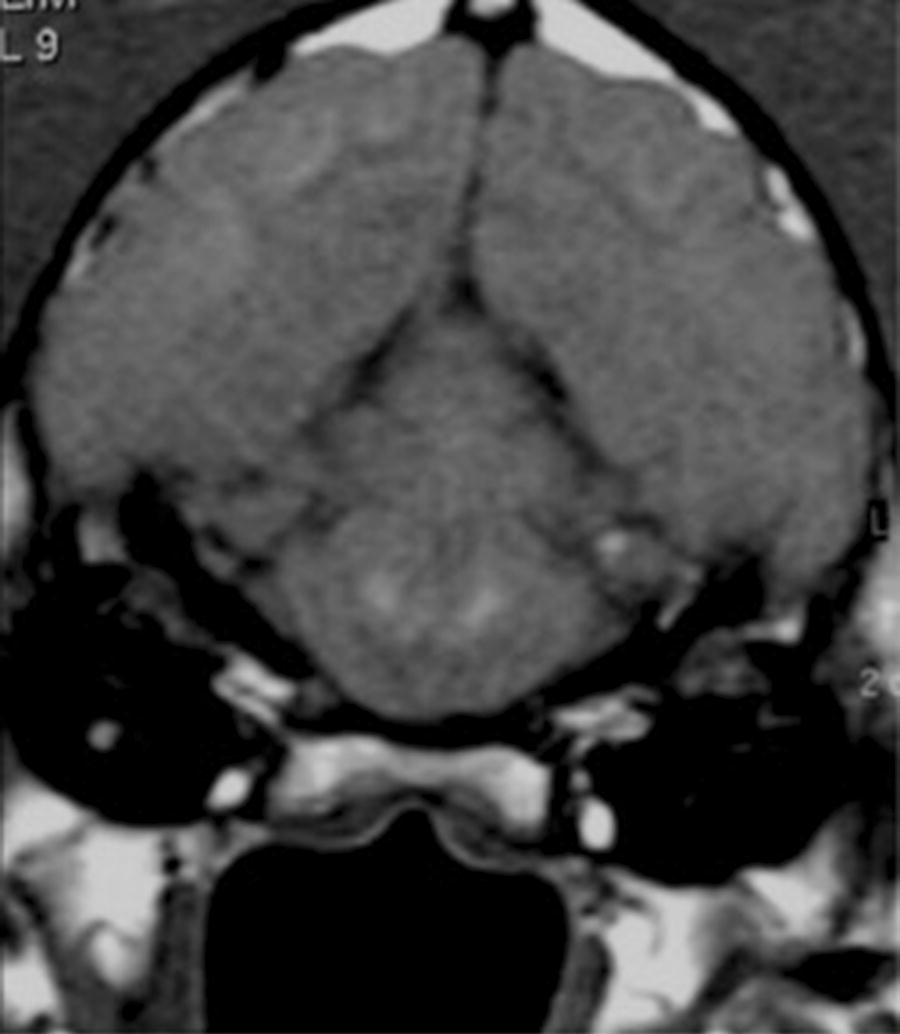
Brain MRI coronal T1 weighted image (post contrast) revealing enhancement in paracentral pontine lesions

Although this investigation was a study of imaging, autopsies were performed on 5 of the 6 dogs who had developed clinical deterioration after correction. Two of the dogs were sacrificed, one clinically normal on day 9 after correction and one ataxic, on day 11 (see Fig. 2 for MRI of this dog). The other autopsies were performed on 4 dogs who had died overnight; when last observed two had been ataxic at days 3 and 4 and two had been stuporous until days 6 and 7 (see Fig. 1 for MRI of one of the stuporous dogs). On microscopy, the lesions seen on imaging showed noninflammatory myelin damage, gliosis and relative sparing of neurons as previously observed [5].

In no case did any MRI sequence show lesions before diffusion weighted imaging did. In 2 dogs diffusion weighted change occurred before any other imaging sequence showed the disease. In some cases diffusion-weighted imaging (DWI) hyperintensity worsened in size and brightness over 2–3 days. In one case Flair images also showed the lesions on the first day of DWI change. In only 3 dogs did gadolinium enhancement of lesions occur on the first day of lesion detection by DWI. In another 3 dogs the enhancement was delayed. The dog which never showed enhancement lived only until day 4. When there were DWI positive lesions evident in multiple territories, it was common for only some of them to show gadolinium enhancement. Eventually lesions were seen on Flair sequences in 5 of the 6 dogs (the exception was a dog who lived only 1 day after the first appearance of myelinolysis on DWI). T_2_ weighted change appeared later in the 3 longest lived dogs.

In a given dog, lesions did not all appear at the same time. Locations for early lesions were the insular and opercular areas (4 dogs) (Fig. 1), pericallosal area (3 dogs), midbrain (3 dogs), and thalamus (3 dogs). Pontine disease (1 dog) was only detected later (day 4) as were cerebellar (2 dogs) and putamenal (2 dogs) lesions (Fig. 2). In the single case of pontine involvement, the disease was not maximal centrally. Instead there were symmetrical paracentral lesions (Fig. 2).

After correction of hyponatremia 5 of the 7 animals developed both neurologic deterioration and brain lesions by MRI. One animal showed clinical deterioration but no lesions by the time of death on day 2. One dog showed lesions but no clinical manifestations. In sum 6 of 7 dogs showed overt neurologic worsening after correction of hyponatremia. In all dogs, which lived at least until day 3, myelinolysis was visible on MRI. The first appearance of MRI change came between days 2 and 5.

### Discussion

The MRI findings in experimental myelinolysis may help us care for patients and evaluate theories about the human disease.

Although there are characteristic brain regions affected by myelinolysis, the combination of affected territories is not uniform from dog to dog. In most dogs the pons showed no disease. In some dogs the thalamus, caudate and midbrain were affected and in others they were not. Only one dog showed disease of the putamen. In other words one pattern of territories can be affected in one animal and different pattern of territories in another.

The subject of regional vulnerability is of particular interest in the pons. Myelinolysis was first discovered in the central pons of autopsied humans [1]. Much speculation followed about what factors predisposed the central pons to the disorder. One dog in this study developed symmetric bilateral pontine rather than central pontine disease. Rarely this pattern of pontine involvement is seen in humans. Thus, whatever features of the pons renders it vulnerable is not necessarily peculiar to the midline. It can be paracentral.

In a particular dog the lesions of myelinolysis did not appear simultaneously on MRI scans. For example, pontine and cerebellar lesions became visible later than cortical and thalamic ones did. A similar delay in the appearance of the pontine lesion has been reported in 3 human cases [6, 7]. The different territorial latencies in a given dog imply differences between animals in a regions’ sensitivity to the disease process.

In myelinolysis there is a complex relationship of the onset of clinical manifestations to appearance of MRI changes. In 3 dogs neurologic deterioration preceded imaging change. There is ample precedent for this sequence in humans [3]. The opposite sequence, the appearance of MRI lesions before evident clinical deterioration also occurred in other dogs, perhaps due to clinical silence of small lesions. Seemingly asymptomatic lesions have been found in humans [1]. However the neurological exam of the dog is not particularly sensitive. The changes in cognition and personality detectable in the human are difficult to recognize in the experimental animal. It is likely that this difficulty in the examination of higher functions accounts for some of the apparent lack of parallelism of clinical and MRI changes in the dog.

Not everything happens simultaneously. Time elapses between the rise in serum sodium and the clinical deterioration. Often there is lag between the appearance of neurologic worsening and the detection of lesions by MRI. Once lesions appear they evolve over days of observation. A satisfactory theory of pathogenesis must explain these latencies.

Others refer to myelinolysis as osmotic demyelination [8]. Exactly how the putative water shift in the brain occurs, how it is related to the latencies mentioned, how it preferentially affects certain cell types and brain regions and why this vulnerability is not the same in all dogs must be explained by any theory of pathogenesis.

It is unknown whether blood barrier breakdown is a critical event in the development of myelinolysis. In 3 dogs gadolinium enhancement of lesions occurred on the first day that lesions were detected. However, some dogs did not show gadolinium enhancement of lesions on the first day that they were detected. In fact, one of the dogs never showed gadolinium enhancement. When gadolinium enhancement did occur, it persisted for days. Because blood brain barrier breakdown (as detected by intravenously injected gadolinium crossing into lesions) was not seen in some dogs, it may be an epiphenomenon rather than an essential early event in myelinolysis.

It is clear from this discussion that we are far from understanding the pathogenesis of myelinolysis. However, these experiments allow us to draw some practical conclusions. Because clinical decline, more often than not, precedes imaging change of myelinolysis the neuroradiologist cannot always see the disease in its early phase. This is important information because it may be beneficial to relower sodium after excessive correction of hyponatremia [8]. Absence of MRI change should not discourage appropriate treatment if myelinolysis is suspected.

### Conclusion

When imaging evidence of myelinolysis does appear, it first occurs on DWI. As a generalization, gadolinium enhancement, flair image change and T_2_ weighted image abnormality appear sequentially.

## Limitations

The authors extrapolate the relevance of the study results using experimental myelinolysis in dogs for theories of pathogenesis and for diagnosis of patients. As mentioned previously, in humans, prospective study of the disease by frequent, sequential scans is not practical and the canine myelinolysis model has been shown to best resembles the human disease both clinically and pathologically [5].

## Abbreviations

MRI: magnetic resonance imaging
DWI: diffusion-weighted imaging
